# Xylose-induced tricarboxylic acid cycle activation resensitizes gentamicin-resistant *Escherichia coli*

**DOI:** 10.1128/aem.00731-26

**Published:** 2026-06-12

**Authors:** Ling Chen, Sufang Kuang, Chenyu Bao, Wenjing Fu, Siying Lin, Tao Yuan

**Affiliations:** 1 Jiangxi Province Key Laboratory of Natural and Biomimetic Drugs Research, College of Pharmacy, Jiangxi Normal University 12642, Nanchang, China; 2 College of Life Sciences, Jiangxi Normal University 12642, Nanchang, China; Kyoto University, Kyoto, Japan

**Keywords:** *Escherichia coli*, xylose, gentamicin, TCA cycle, reactivating

## Abstract

The escalating antibiotic resistance crisis requires innovative strategies to revitalize existing antibiotics. Here, we report that xylose, a food-grade sugar poorly metabolized by humans but readily utilized by bacteria, acts as a potent metabolic adjuvant to restore gentamicin efficacy against bacteria. Using a laboratory-evolved gentamicin-resistant *E. coli* strain, we showed that xylose enhances gentamicin-mediated killing in a dose- and time-dependent manner, achieving a 200-fold reduction in viability with a synergy score 25.25. This synergistic bactericidal effect was also confirmed in multidrug-resistant and carbapenem-resistant bacteria and a mouse infection model, boosting survival rates by 30%. Metabolomics and pathway analyses revealed that xylose induced a global metabolic shift, upregulating pyruvate and succinate as biomarkers and activating the tricarboxylic acid (TCA) cycle. Exogenous addition of pyruvate and succinate also contributed to the bactericidal effect of gentamicin. This is accompanied by increased activity of key TCA enzymes (PDH, α-KGDH, SDH, MDH), elevated NADH and ATP levels, and enhanced proton motive force (PMF). However, when TCA cycle enzymes α-ketoglutarate dehydrogenase or succinate dehydrogenase were inhibited via gene knockout or sodium malonate, or when PMF was dissipated by carbonyl cyanide m-chlorophenylhydrazone (CCCP), the synergistic bactericidal effect was abolished. Xylose treatment increased intracellular gentamicin accumulation by 2.34-fold, an effect that was also blocked by TCA cycle or PMF inhibition. Our study established xylose as a bacteria-selective adjuvant that revitalizes gentamicin by reactivating TCA cycle dependent drug uptake, offering a potential strategy to combat antibiotic resistance.

**IMPORTANCE:**

This work provided a critical advance in the fight against antibiotic resistance by transforming a common food-grade sugar into a targeted therapeutic tool. Unlike conventional antibiotics or previously reported adjuvants, xylose is uniquely selective: it is efficiently utilized by bacteria but poorly metabolized in humans, thereby minimizing off-target effects. We demonstrated for the first time that xylose reversed resistance by reactivating the silenced TCA cycle in resistant bacteria, going beyond mere substrate provision to restore a critical metabolic function. This mechanism not only revitalized the efficacy of gentamicin but also established a new strategy, exploiting the metabolic differences between host and pathogen to develop “pathogen-focused” adjuvants.

## INTRODUCTION

Antibiotic resistance currently poses one of the greatest threats to human health. The widespread use and environmental residues of antibiotics have accelerated the emergence of resistant bacteria (1). Antibiotic resistance is primarily mediated through four mechanisms, including decreased membrane permeability, increased efflux pumps, reduced target binding, and enzymatic drug modification or destruction (2). Given these resistance mechanisms, the efficacy of existing antibiotics has been severely compromised, rendering them increasingly inadequate against resistant pathogens (3). Therefore, there is an urgent need to develop novel strategies to restore or enhance the effectiveness of conventional antibiotics (4).

To address the challenges posed by antibiotic resistance mechanisms, a promising approach is to increase intracellular antibiotic concentrations by enhancing membrane permeability or reducing antibiotic efflux, which is essential for repurposing conventional antibiotics (2, 5). Aminoglycoside antibiotics, whose action relies on bacterial active uptake, often face resistance due to an uptake barrier caused by pathogen metabolic downregulation (6). Excitingly, recent studies have demonstrated that metabolic reprogramming can overcome this barrier, effectively promoting the uptake of aminoglycoside antibiotics (1, 2, 5, 7–11). Such effects have been observed when combining sodium formate with micronomicin in drug-resistant *E. coli* (12), uracil with gentamicin in methicillin-resistant *Staphylococcus aureus* (MRSA) (13), nicotinamide adenine dinucleotide (NADH) with neomycin in drug-resistant *E. tarda* (14), and fructose with gentamicin in antibiotic-resistant *Salmonella enteritidis* (15). Through metabolic reprogramming, these drug-resistant bacteria become sensitive to killing by aminoglycoside antibiotics (micronomicin, gentamicin, or neomycin) (16). However, all metabolites reported to date that promote aminoglycoside uptake are metabolizable by both bacteria and humans; therefore, an adjuvant with selective microbial metabolism would offer a more targeted strategy.

D-Xylose, a low-glycemic monosaccharide widely used in the food industry, represents a unique opportunity. It offers an advantage as a metabolic adjuvant because it is poorly metabolized by the human body yet readily utilized by various microbes. However, its potential role as an antibiotic potentiator, and the mechanistic basis for such an effect, remains completely unexplored. Here, we hypothesized that xylose can reprogram the metabolism of drug-resistant *E. coli*, break their defensive lethargy, and promote the uptake of gentamicin. Through integrated *in vitro* and *in vivo* studies, combined with metabolomics and functional assays, we aimed to (i) confirm the bactericidal effect of xylose combined with gentamicin against laboratory-evolved and clinically resistant strains *in vitro* and *in vivo* models, (ii) map the global metabolic alterations induced by xylose to identify the key pathway involved, and (iii) validate the mechanism by which xylose promotes intracellular antibiotic accumulation. Here, we provide a mechanistic rationale and translational strategy for employing xylose as an adjuvant to combat antibiotic resistance.

## RESULTS

### Xylose promoted gentamicin to eradicates high-burden gentamicin-resistant and multidrug-resistant Gram-negative pathogens

To model severe infections with a high gentamicin resistant bacteria loads with approximately 10^8^ CFU/mL and assess xylose’s rapid-response effectiveness, we first conducted a comprehensive phenotypic analysis of gentamicin resistant bacteria (ECO-R_GEN_) derived from susceptible *Escherichia coli* K12 BW25113 (ECO-S) through stepwise gentamicin passage. The results revealed that ECO-R_GEN_ exhibited a 32-fold increase in gentamicin minimum inhibitory concentration (MIC), a growth profile consistent with resistance, reduced susceptibility in disk diffusion assays, and impaired motility compared to ECO-S (Fig. S1), confirming a distinct phenotypic and potential metabolic state. Whole-genome sequencing of the 3 ECO-R_GEN_ strains identified mutations in 49 genes that were commonly altered across all three isolates and were involved in diverse functional categories (Table S1). These genes were involved in central carbon metabolism including *sucA*, *aceE*, *ppc*; transcription/translation including *rpoB*, *rpoC*, *dnaE*; cell division *mukB*, efflux pump *mdtC*, outer membrane transport *fepA*, and stress responses *katG*, indicating a complex, pleiotropic genetic basis for gentamicin resistance.

**Fig 1:**
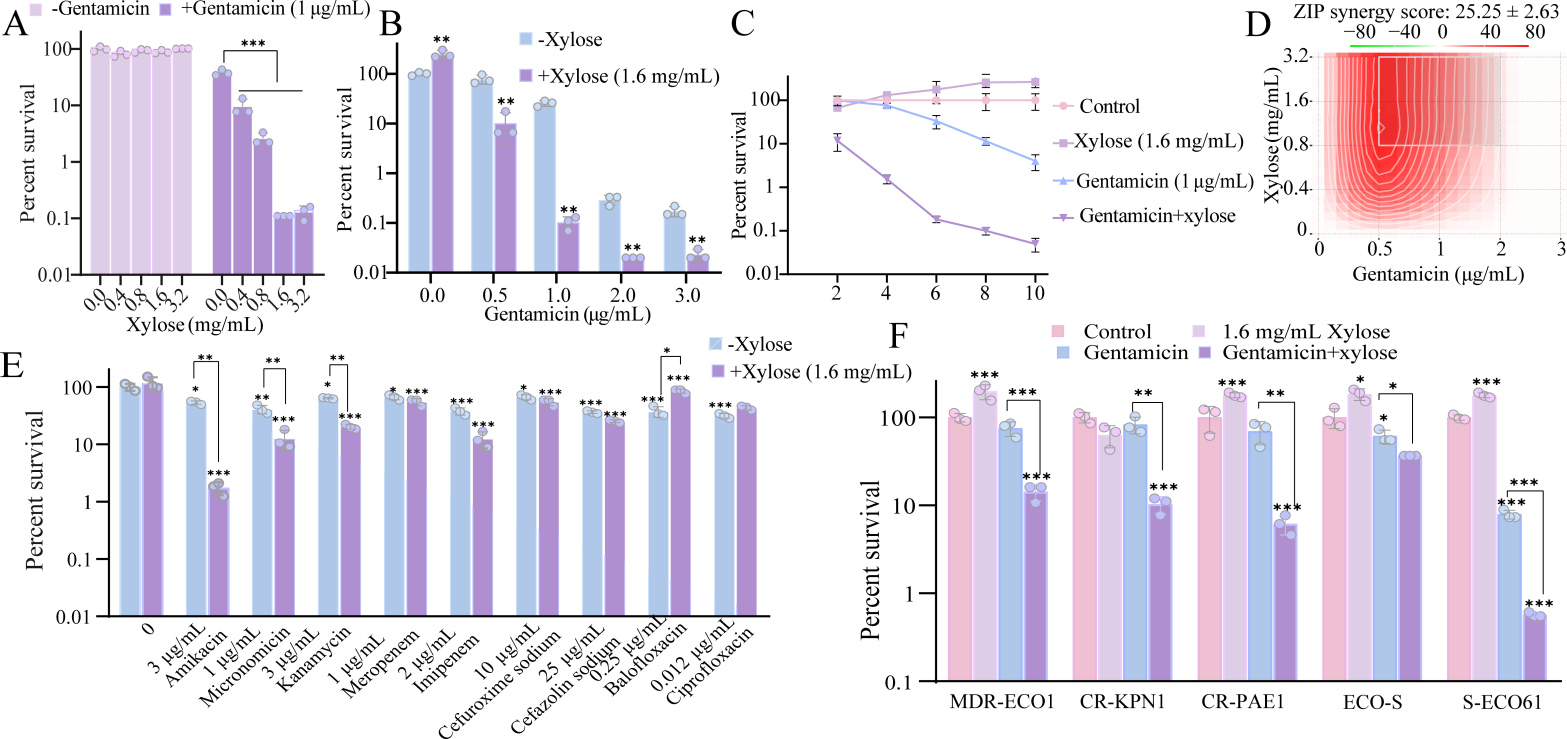
Antibacterial efficacy by gentamicin in the presence of xylose. (**A**) Percent survival of ECO-R_GEN_ at the indicated concentrations of xylose plus 1 μg/mL gentamicin using M9 medium for 6 h. (**B**) Percent survival of ECO-R_GEN_ at the indicated concentrations of gentamicin plus 1.6 mg/mL xylose using M9 medium for 6 h. (**C**) Percent survival of ECO-R_GEN_ for different incubation times with 1.6 mg/mL xylose and 1 μg/mL gentamicin using M9 medium. (**D**) Synergy plot analysis of the percent survival for xylose and/or gentamicin by SynergyFinder (https://synergyfinder.org). (**E**) Percent survival of ECO-R_GEN_ in the presence of other aminoglycoside antibiotics (amikacin, micronomicin, and kanamycin), carbapenems (meropenem, imipenem), cephalosporins (cefuroxime sodium and cefazolin sodium), and fluoroquinolones (balofloxacin, ciprofloxacin) plus 1.6 mg/mL xylose using M9 medium for 6 h. (**F**) Percent survival of clinically isolated multidrug-resistant bacteria or susceptible bacteria in the presence of 1.6 mg/mL xylose and/or gentamicin using M9 medium for 6 h. The concentrations of gentamicin used for MDR-ECO1, CR-KPN1, CR-PAE1, ECO-S, S-ECO61 were 40, 100, 10, 0.5, and 1 μg/mL, respectively. All experiments were conducted with three independent biological replicates, and data (**A–C, E and F**) were expressed as mean ± SD. Statistical analysis was performed using two-way ANOVA followed by Tukey’s correction to determine significant differences between groups (*P < 0.05, **P < 0.01, ***P < 0.001). In panel E, "star(s)" above treatment with drug alone is in comparison to survival of the strain without the drug.

To overcome this resistance, we tested xylose as an adjuvant. Based on the concentrations of glucose, fructose, and xylose reported in the previous reports (15, 17–19), we conducted a gradient experiment on xylose concentration. Xylose potentiated gentamicin-mediated killing of ECO-R_GEN_ in a xylose or gentamicin-dependent and time-dependent manner (Fig. 1A through C). Synergy between xylose and gentamicin was quantified using the Zero Interaction Potency (ZIP) model in Synergy Finder. The overall synergy score was 25.25 (Fig. 1D), significantly above the threshold of 10. Spot dilution assay at 6 h showed that xylose plus gentamicin reduced bacterial viability by approximately 200-fold relative to the initial inoculum at 0 h, whereas no reduction was observed in the control or xylose-alone groups and only a modest reduction occurred with gentamicin alone (Fig. S2), confirming strong synergistic bactericidal activity between the two compounds. When xylose was combined with other aminoglycoside antibiotics (amikacin, micronomicin, and kanamycin), carbapenems (meropenem, imipenem), and cephalosporins (ceftriaxone sodium, cefazolin sodium), quinolones (balofloxacin, ciprofloxacin), the survival rate of xylose combined with aminoglycoside antibiotics was lower than with the aminoglycoside antibiotics alone (Fig. 1E; Fig. S3A), and synergy effect was demonstrated (Fig. S3B). However, no potentiating effect of xylose was observed on other non-aminoglycoside antibiotics. Since the mechanisms of action of these non-aminoglycoside antibiotics differ from that of aminoglycosides, which rely on the proton motive force (PMF) (20, 21), the potentiating effect of xylose on antibiotic bactericidal activity was primarily applicable to aminoglycoside antibiotics. Subsequently, clinically isolated multidrug-resistant *Escherichia coli* 1 (MDR-ECO1), carbapenem-resistant bacteria *Klebsiella pneumoniae* 1 (CR-KPN1), and *Pseudomonas aeruginosa* 1 (CR-PAE1) (Table S2) were employed to evaluate the potentiating effect of xylose. MDR-ECO1 was resistant to at least three classes of antibiotics tested but sensitive to meropenem, and/or imipenem; CR-KPN1 and CR-PAE1 were resistant to at least three classes of antibiotics tested (including gentamicin) as well as to meropenem and/or imipenem. The survival rate with the combination of gentamicin and xylose was lower than that with gentamicin alone (Fig. 1F). Meanwhile, xylose also enhanced gentamicin-mediated killing of the model susceptible strain ECO-S and a clinically isolated susceptible strain ECO-S61 (Fig. 1F). This indicated that the effect of xylose was not limited to the gentamicin resistance mechanisms. To investigate whether xylose enhances gentamicin killing in the presence of other carbon sources, we replaced sodium acetate with varying concentrations of glucose. The synergistic effect of xylose was completely abolished at high glucose concentrations, whereas it could be manifested at low glucose concentrations (Fig. S4). As previously reported, in the presence of both glucose and xylose, the CcpA/HPr-P complex inhibits the transcription of the *xylA/B* gene (22), suggesting that glucose interferes with the utilization of xylose. Together, these findings demonstrated that xylose effectively enhanced the bactericidal activity of gentamicin against both laboratory-evolved and clinically relevant bacteria, regardless of whether they are susceptible or drug-resistant.

### Xylose induced metabolic phenotypic changes in ECO-R_GEN_

To uncover the metabolic mechanisms underlying the enhanced gentamicin-mediated bactericidal effect of exogenous xylose, we utilized a GC-MS-based metabolomics analysis to compare the metabolic profiles of ECO-R_GEN_ with or without xylose treatment. A total of 16 data sets were generated from four biological replicates, each with two technical replicates. Total ion current (TIC) chromatograms provided a comprehensive overview of all metabolites present in each sample, and significant differences were observed between the xylose group and control group (Fig. S5A). The correlation coefficient for technical replicates demonstrated an *R*² value of 0.9967 (Fig. S5B). A total of 72 metabolites were identified in each sample (Fig. S5C). The metabolic heatmap depicted the distribution of metabolites, categorized as carbohydrates, amino acids, lipids, nucleotides, and other categories (Fig. S5D). Comparison of the control and xylose groups revealed 60 differentially abundant metabolites were identified in the xylose group (Fig. S6A). *Z*-score varied between −5.94 and 127.16 in the xylose group (Fig. S6B), with 57 and 3 differential metabolites were upregulated and downregulated, respectively. The three most significantly increased metabolites were xylose, succinic acid, and acetic acid, whereas sorbose, sucrose, and uracil showed the most pronounced decreasing trends. These findings suggest that xylose induces a differential metabolome. Further classification and statistical analysis of the differentially abundant metabolites showed that carbohydrates accounted for the highest proportion with 40% (Fig. S6C), suggesting broad metabolic remodeling in response to xylose supplementation.

### Identification of potential biomarkers and differential metabolic pathways induced by xylose in ECO-R_GEN_

**Fig 2:**
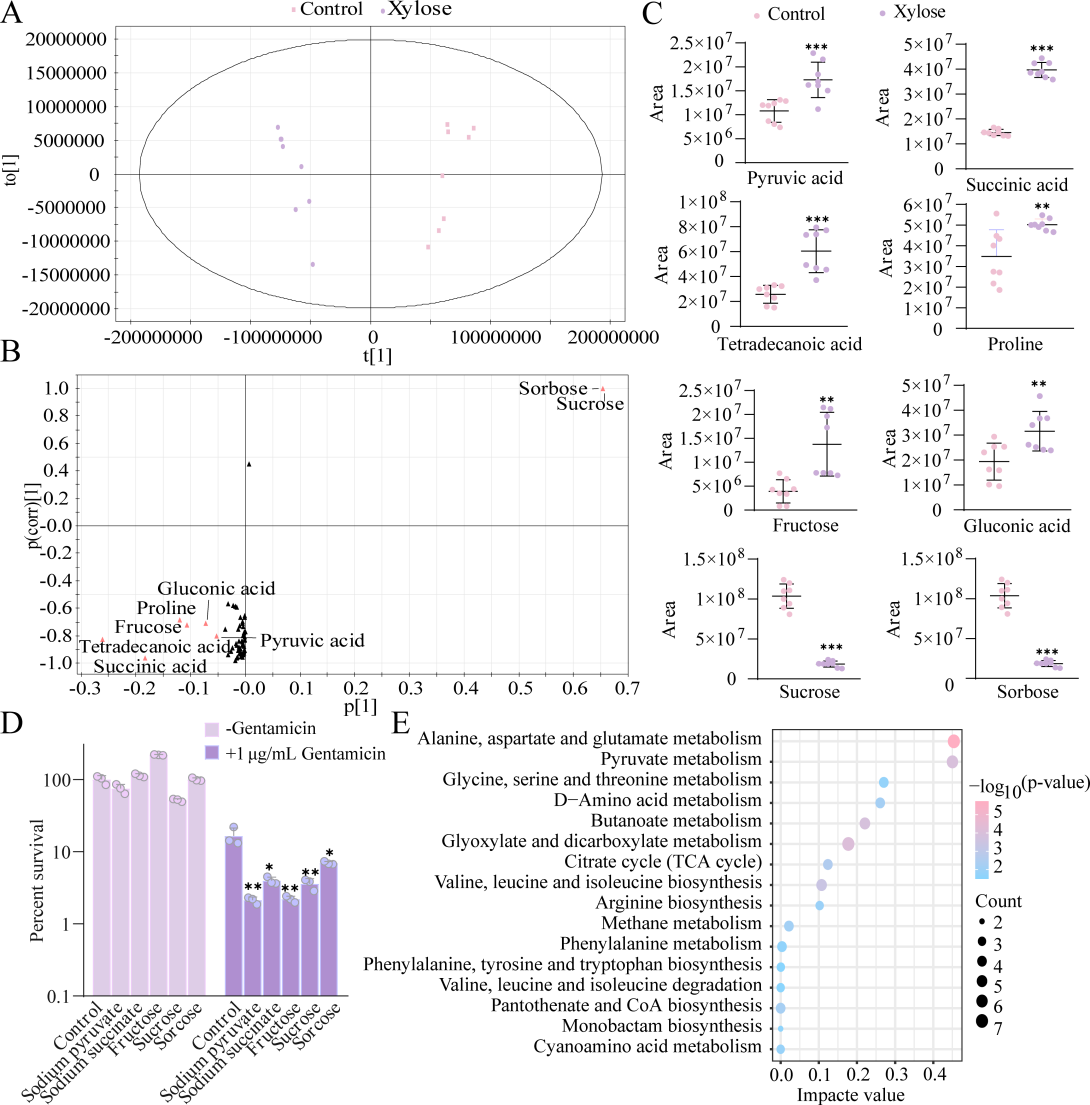
Identification of potential metabolite biomarkers and key metabolic pathways induced by xylose. (**A**) OPLS-DA of metabolic profile data for ECO-R_GEN_ with or without xylose. (**B**) S-plot generated from panel A. (**C**) The scatter plot of metabolite biomarkers. (**D**) Percent survival of ECO-R_GEN_ for 6 h with 5 mM potential metabolite biomarkers and/or 1 μg/mL gentamicin using M9 medium. (**E**) KEGG pathways with *P* < 0.05 were visualized. Experiments shown in panels A, B, C, and E were conducted with eight identical copies (four biological replicates, each with two technical replicates), while the experiment shown in panel D was conducted with three biological replicates; data in panels C and D are expressed as mean ± SD. Statistical analysis was performed using one-way analysis of variance (ANOVA) to determine significant differences between groups (**P < 0.01, ***P < 0.001).

To better distinguish the metabolic states of the control and xylose groups, orthogonal partial least squares discriminant analysis (OPLS-DA) was performed. As shown in Fig. 2A, component t[1] clearly distinguishes the xylose group from the control group, indicating a substantial influence of xylose on the metabolic state of drug-resistant bacteria. The S-plot derived from the OPLS-DA model was analyzed. In the plot of predictive correlation between p[1] and p(corr)[1], the red triangles represented differential metabolites with larger weights (< −0.05 or >0.05) and higher relevance (< −0.5 or >0.5). Eight metabolites (pyruvic acid, succinic acid, fructose, gluconic acid, tetradecanoic acid, proline, sucrose, sorbose) were identified as potential biomarkers (Fig. 2B). Among them, six metabolites (pyruvic acid, succinic acid, fructose, gluconic acid, tetradecanoic acid, proline) were upregulated, and two metabolites (sucrose, sorbose) were downregulated in response to xylose (Fig. 2C). Notably, the two upregulated biomarkers include pyruvate and succinate, which link xylose metabolism to central carbon pathways such as pyruvate metabolism and the tricarboxylic acid (TCA) cycle, while fructose, sucrose, and sorbose were associated with sugar metabolism and energy provision for bacteria. To investigate whether the metabolic changes of these five biomarkers are functionally linked to gentamicin potentiation, each metabolite was added to ECO-R_GEN_ cultures at concentrations consistent with those observed in the metabolomics profiles, and bacterial survival was assessed following gentamicin treatment. Exogenous supplementation of sodium pyruvate, sodium succinate, fructose, sucrose, and sorbose significantly enhanced gentamicin-mediated killing compared with gentamicin alone (Fig. 2D). These observations suggest that the relevant metabolic pathways warrant further analysis. Investigation of the differential metabolic pathways is critical for further pinpointing the specific processes altered by xylose. Enrichment analysis revealed 16 significantly altered metabolic pathways (Fig. 2E). The top three pathways ranked by impact value were alanine, aspartate, and glutamate metabolism; pyruvate metabolism; and glycine, serine, and threonine metabolism. Notably, the top three pathways include two (pyruvic acid and succinic acid), two (pyruvic acid and succinic acid), and one (pyruvic acid) of the above-mentioned biomarkers, respectively. This established a direct connection between pyruvate metabolism and the TCA cycle, thus creating a continuous metabolic flux. Prominent enhancements were observed in energy metabolism and carbohydrate metabolism, directly related to the rapid entry of xylose as an exogenous carbon source into central carbon metabolism, including pyruvate metabolism and the TCA cycle.

### Xylose stimulates carbon flux and transcriptional activation of central metabolic pathways

**Fig 3:**
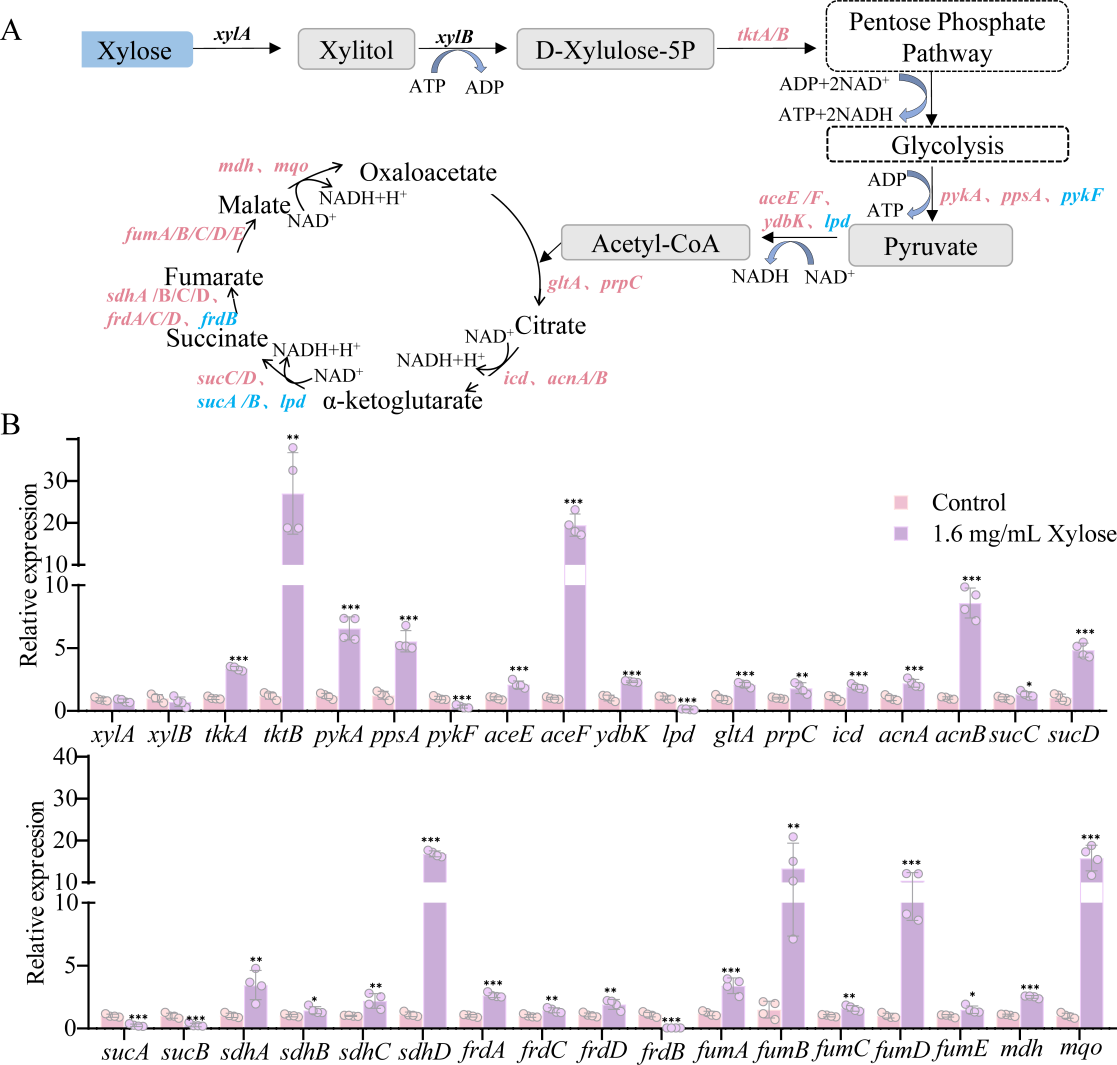
Analysis of xylose-induced differential metabolic pathways and flux. (**A**) Xylose metabolic pathway. (**B**) The gene expression in ECO-R_GEN_ with or without 1.6 mg/mL xylose. All experiments were carried out with four biological replicates, and data shown in panel B are expressed as mean ± SD. Statistical analysis was performed using one-way analysis of variance (ANOVA) to determine significant differences between groups (*P < 0.05, **P < 0.01, ***P < 0.001).

To delineate the metabolic flux directly driven by xylose, we analyzed its pathway and the expression of key genes (Fig. 3A). Gene expression analysis by quantitative real-time PCR (qRT-PCR) revealed widespread upregulation of genes involved in these processes (Fig. 3B). Among the 35 genes examined, 28 were upregulated and only 5 were downregulated. This coordinated transcriptional activation indicated that xylose effectively channels carbon flux through glycolysis and pyruvate metabolism, ultimately converged into the TCA cycle, thereby elevated the metabolic state of the resistant bacteria. This observation was also consistent with a previous report showing downregulation of the TCA cycle following gentamicin resistance (23), raising the possibility that xylose-induced metabolic stimulation could counteract resistance-associated metabolic suppression. However, direct causal evidence linking these transcriptional changes to antibiotic sensitization requires further functional validation. Thus, the relationship between carbon influx into the TCA cycle and restored antibiotic efficacy warrants mechanistic investigation.

### Xylose promoted gentamicin uptake by reactivating the TCA cycle

**Fig 4:**
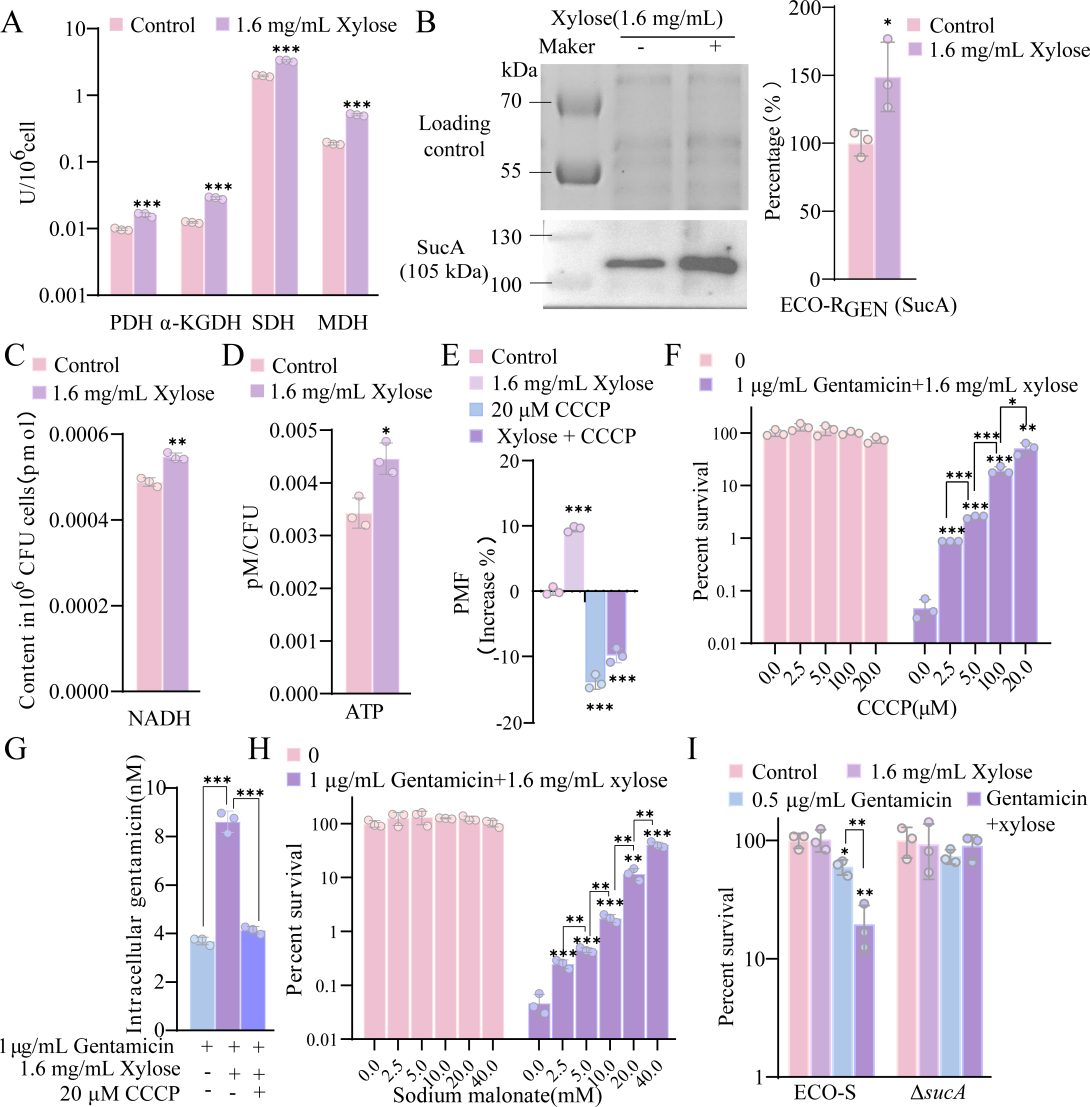
TCA cycle promoted by exogenous xylose. (**A**) Enzymes activity analysis of the TCA cycle in ECO-R_GEN_ with or without 1.6 mg/mL xylose. (**B**) SucA protein expression level in ECO-R_GEN_ with or without 1.6 mg/mL xylose by western blot. (**C** and **D**) NADH level (**C**) or ATP level (**D**) of ECO-R_GEN_ with or without 1.6 mg/mL xylose. (**E**) PMF of ECO-R_GEN_ incubated for 6 h in M9 medium with 1.6 mg/mL xylose and/or 20 μM CCCP. (**F**) Percent survival of ECO-R_GEN_ in the presence of 1.6 mg/mL xylose plus 1 μg/mL gentamicin with indicated concentrations of CCCP. (**G**) Oxford cup method for intracellular gentamicin content of ECO-R_GEN_ strains in the presence of 1 μg/mL gentamicin with or without 1.6 mg/mL xylose. (**H**) Percent survival of ECO-R_GEN_ in the presence of 1.6 mg/mL xylose plus 1 μg/mL gentamicin with indicated concentrations of sodium malonate. (**I**) Percent survival of ECO-S and its *sucA* gene knockout mutant (Δ*sucA*) in the presence of 1.6 mg/mL xylose and/or 0.5 μg/mL gentamicin in M9 medium for 6 h incubation. All experiments were carried out with three biological replicates, and data shown in panels A and C to I are expressed as mean ± SD. Statistical analysis was performed using two-way ANOVA followed by Tukey’s correction to determine significant differences between groups (*P < 0.05, **P < 0.01, ***P < 0.001).

To elucidate how xylose overcomes the uptake barrier in resistant *E. coli*, we investigated whether carbon influx into the TCA cycle correlates with enhanced intracellular accumulation of gentamicin. The activities of four key TCA cycle enzymes: pyruvate dehydrogenase (PDH), α-ketoglutarate dehydrogenase (α-KGDH), succinate dehydrogenase (SDH), and malate dehydrogenase (MDH) were significantly elevated in ECO-R_GEN_ following xylose treatment (Fig. 4A). This enzymatic activation was corroborated at the protein level by increased expression of SucA, a core subunit of α-KGDH, in the xylose-treated group (Fig. 4B). The enhanced TCA cycle flux led to a substantial rise in cellular energy charge and reducing equivalents. Specifically, intracellular NADH and ATP levels increased markedly upon xylose supplementation (Fig. 4C and D). It may be linked to increased PMF, which is critical for aminoglycoside uptake as previously described (10). Accordingly, we measured the PMF levels and found that the PMF significantly increased after xylose addition. Moreover, the PMF dissipator carbonyl cyanide m-chlorophenylhydrazone (CCCP) effectively reduced the PMF in the xylose-treated group (Fig. 4E). Correspondingly, CCCP abolished the synergistic killing effect in a dose-dependent manner (Fig. 4F). To directly connect this mechanism to drug uptake, we quantified intracellular gentamicin accumulation. Xylose treatment resulted in an approximately 2.34-fold increase in gentamicin content, an effect that was completely abolished by co-treatment with CCCP (Fig. 4G). We then attempted two methods to block this pathway. Addition of sodium malonate, a competitive inhibitor of SDH, dose-dependently rescued bacterial survival from the xylose-gentamicin combination (Fig. 4H). Furthermore, knockout of the *sucA* gene in ECO-S also reversed the synergistic killing effect of the xylose-gentamicin combination (Fig. 4I). These data demonstrated that xylose promoted gentamicin uptake primarily by reactivating the TCA cycle, thereby driving increased intracellular accumulation of the antibiotic and enhanced its bactericidal effect.

### Xylose boosted the activity of gentamicin to eradicate resistant *E. coli* in mice

**Fig 5:**
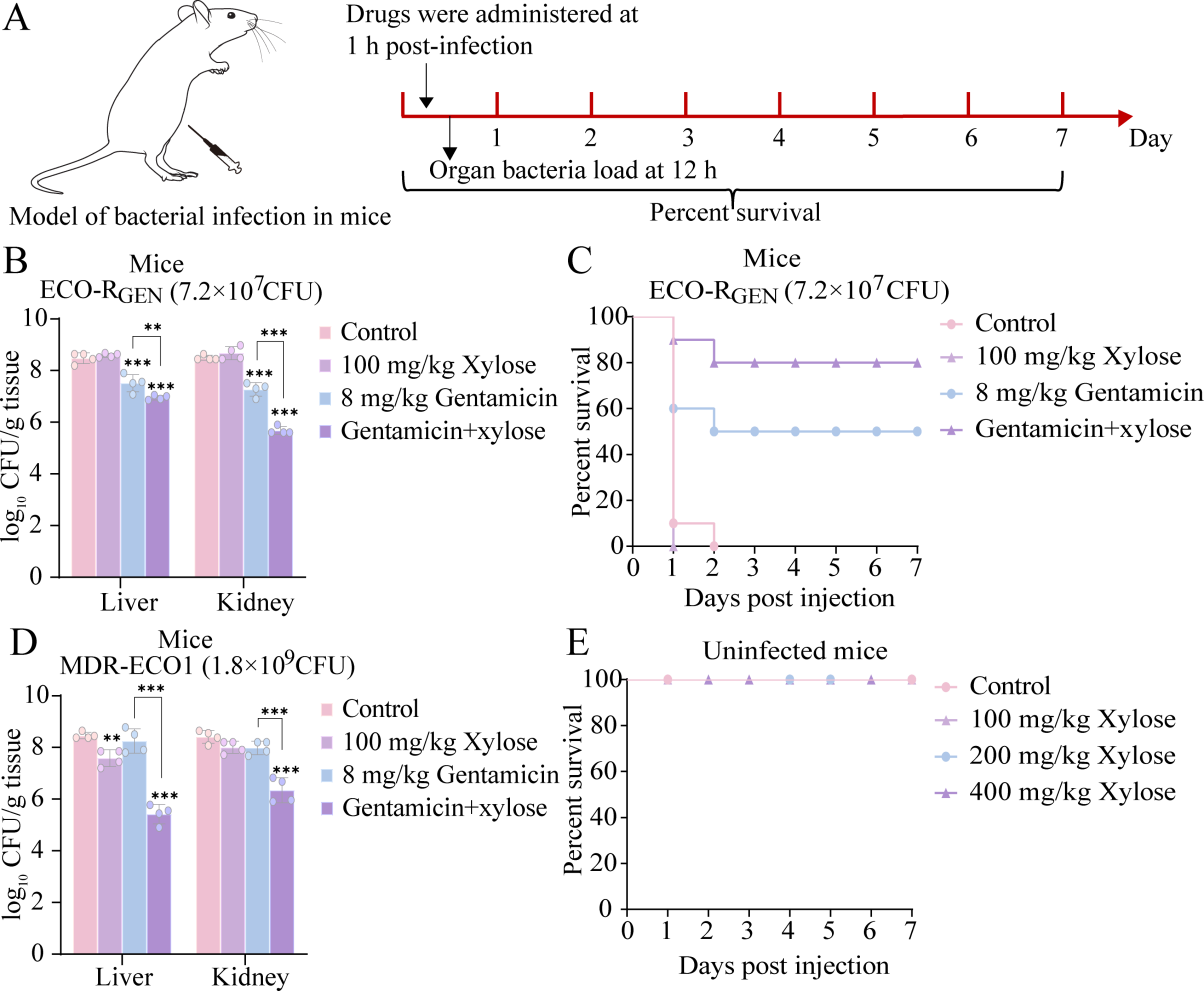
The impact of xylose on the anti-infective ability of gentamicin in mice. (**A**) Mouse bacterial infection model. (**B**) Determination of organ bacterial count in mice infected with ECO-R_GEN_ and treated with gentamicin and/or xylose. (**C**) Survival rate of mice infected with ECO-R_GEN_ and treated with gentamicin and/or xylose. (**D**) Determination of organ bacterial count in mice infected with MDR-ECO1 and treated with gentamicin and/or xylose. (**E**) Survival rate of uninfected mice after injection of high doses of xylose. Experiments shown in panels C and E were conducted with 10 biological replicates under identical conditions, while experiments shown in panels B and D were performed with four biological replicates; data shown in panels B and D are expressed as mean ± SD. Statistical analysis was performed using two-way ANOVA followed by Tukey’s correction to determine significant differences between groups (**P < 0.01, ***P < 0.001).

The effect of xylose combined with gentamicin on eradicating drug-resistant *Escherichia coli* was further investigated in a mouse model. Based on a previously reported oral dose of xylose at 100 mg/kg (24), we administered xylose at this dose. Mice were infected with a lethal dose of ECO-R_GEN_ and treated with saline, xylose alone (100 mg/kg, p.o.), gentamicin alone (8 mg/kg, i.m.), or their combination. To assess therapeutic efficacy, bacterial loads in the liver and kidneys were measured 12 h post-treatment (Fig. 5A). The combination therapy resulted in a 4.4-fold and 42-fold reduction in liver and kidney bacterial counts compared to the gentamicin group, respectively (Fig. 5B). This enhanced clearance directly correlated with a survival benefit over the following days; mice receiving the combination therapy exhibited a 30% higher survival rate than those treated with gentamicin alone (Fig. 5C). When the clinically isolated MDR-ECO1 was used for infection, the combination therapy reduced bacterial loads in the liver and kidneys by 806-fold and 33-fold compared with the gentamicin group, respectively (Fig. 5D). Additionally, healthy mice administered a gradient of xylose doses (100, 200, and 400 mg/kg, p.o.) showed no mortality in any group (Fig. 5E). These results demonstrated that xylose significantly improved the survival of infected mice by boosting gentamicin-mediated eradication of resistant *E. coli*.

## DISCUSSION

The escalating crisis of antimicrobial resistance has emerged as a global public health emergency (25). This critical situation necessitates the urgent development of innovative strategies to restore or enhance the effectiveness of conventional antibiotics. Metabolic reprogramming can effectively alter the metabolic state of bacteria, thereby increasing the sensitivity of drug-resistant bacteria to conventional antibiotics (2, 5). Metabolites such as sodium formate, uracil, NADH, and fructose have been shown to overcome the antibiotic uptake barrier through metabolic reprogramming, thereby resensitizing bacteria to aminoglycosides including gentamicin, micronomicin, and neomycin (12–15). However, these metabolites can be utilized by both bacteria and humans; therefore, identifying a sensitizer that is metabolically active only in bacteria represents a more targeted strategy (26, 27). This study was conceived to test the hypothesis that xylose, a food-grade sugar with minimal human metabolism, could serve as a metabolic adjuvant to promote gentamicin uptake. Our findings not only confirm this hypothesis but also delineate a mechanistic pathway from xylose to restored antibiotic efficacy.

Here, we demonstrated that xylose synergistically enhanced gentamicin-mediated killing in a dose- and time-dependent manner, achieving up to a 200-fold reduction in bacterial survival *in vitro*. Critically, this potentiation was effective against both laboratory-evolved and clinically isolated multidrug-resistant strains, including carbapenem-resistant *Klebsiella pneumoniae* and *Pseudomonas aeruginosa*, and translated into significantly improved bacterial clearance and survival in murine infection models. This finding extends the potential antibacterial applications of xylose, which was recently shown to serve as a targeting moiety in a D-xylose decorated antimicrobial peptide carbon dot system against multidrug-resistant *E. coli* (28). This finding fulfills the urgent need for strategies that revitalize existing antibiotics against resistant pathogens.

In contrast to many previously reported artificial sweeteners, such as xylitol, saccharin, or others that not only potentiate antibiotic activity but also inhibit bacterial growth (29–31), our study shows that xylose (1.6 mg/mL, ~10.7  mM) itself does not inhibit bacterial growth under the tested conditions, yet it significantly enhances gentamicin-mediated killing. This distinction is important because it suggests that xylose acts primarily as a metabolic reprogramming agent rather than as an additional antibacterial stressor, thereby avoiding the additional selective pressure that might accelerate resistance development. Furthermore, while it is well established that certain carbon sources, such as glucose or fructose (16), can reduce antibiotic tolerance; most of these are also readily utilized by the host. Xylose offers a unique advantage in that it is poorly metabolized by humans but efficiently utilized by bacteria, making it a more selective adjuvant for *in vivo* applications.

We further investigated the mechanism by which xylose promotes the highly efficient bactericidal effect of gentamicin. Our metabolomics and pathway analyses revealed that xylose induced a global metabolic shift, with the most pronounced changes occurring in central carbon metabolism. Pyruvate and succinate, two key intermediates of the TCA cycle, were significantly upregulated and identified as potential biomarkers. Exogenous addition of these metabolites recapitulated the gentamicin-potentiating effect, directly linking the xylose-induced metabolic flux to antibiotic synergy. This finding was consistent with earlier work showing that fumarate, another TCA cycle intermediate, potentiates tobramycin against *Pseudomonas aeruginosa* by fueling the TCA cycle (9). Our study extends this concept by identifying a non-mammalian carbon source that can achieve similar metabolic activation without host competition.

Metabolomic analysis also revealed that xylose induced differential metabolomes in ECO-R_GEN_, with the top three altered metabolic pathways being alanine, aspartate, and glutamate metabolism; pyruvate metabolism; and glycine, serine, and threonine metabolism. Crucially, key biomarkers, particularly pyruvic acid and succinic acid, were enriched within these top pathways, establishing a direct metabolic connection between pyruvate metabolism and the TCA cycle. This observed flux is consistent with the emerging paradigm that central carbon metabolism serves as a critical leverage point for modulating antibiotic susceptibility (2). Similarly, pyruvate activates central carbon metabolism, reverses antibiotic resistance, and enhances the bactericidal activity of gentamicin against drug-resistant *Vibrio alginolyticus* (10). Concurrent upregulation of amino acid, nucleotide, and cofactor metabolism further indicates that xylose broadly stimulates bacterial biosynthetic capacity. This also directly boosted the engine that generates energy (32). Subsequently, qRT-PCR was used to analyze the expression levels of 35 genes involved in energy and carbohydrate metabolism directly related to glycolysis, pyruvate metabolism, and the TCA cycle; of these, 28 were upregulated. This coordinated gene expression demonstrates that xylose effectively channels carbon through glycolysis and pyruvate metabolism into the TCA cycle, thereby elevating the metabolic state of the resistant bacteria. Previous work has shown that gentamicin resistance was accompanied by suppression of central carbon and energy metabolism, including the TCA cycle (23, 33). Our findings revealed that xylose precisely reverses this suppression, reactivating the TCA cycle to regenerate energy. This notion was supported by a separate study in which adaptation of *E. coli* to xylose led to significant upregulation of TCA cycle proteins, correlating with enhanced metabolic activity and sugar utilization (34).

Thus, the influx of carbon into the TCA cycle prompted us to investigate whether it is associated with increased uptake of antibiotics. More importantly, we confirmed that xylose increased the activity of key TCA cycle enzymes (PDH, α-KGDH, SDH, MDH), including an increased in the expression level of SucA, thereby elevating the levels of NADH and ATP. Within the electron transport chain, NADH-driven PMF accelerates aminoglycoside uptake (14, 16, 33). Thus, we further confirmed the role of the TCA cycle in xylose-mediated promotion of the bactericidal effect of gentamicin by increasing uptake. The synergistic effect was abolished by knockout of the *sucA* gene, by the SDH inhibitor sodium malonate and by the PMF uncoupler CCCP. Further experiments indicated that CCCP also prevented the xylose-induced increase in intracellular gentamicin accumulation. Thus, we demonstrated that xylose not only entered the TCA cycle but also reactivated the functionally impaired cycle in resistant bacteria, thereby enhancing antibiotic uptake. Importantly, this effect was specific to aminoglycosides because their uptake depends on PMF, whereas other antibiotic classes (β-lactams, quinolones) rely on different mechanisms. This specificity may reinforce the mechanistic model by which xylose promotes the effect of aminoglycosides.

However, a major concern in the field is whether metabolic adjuvants work only against certain resistance pathways. Our laboratory-evolved resistant strain ECO-R_GEN_ and the three clinical isolates harbor drug resistance to different antibiotics. Despite these diverse resistance genotypes, xylose consistently potentiated gentamicin killing. Moreover, xylose also enhanced gentamicin activity against susceptible strains ECO-S and a clinical susceptible isolate. This suggested that the effect is not a rescue specific to a defective pathway but rather a general enhancement of PMF-dependent uptake that is more impactful when baseline uptake is compromised. These findings were important because they indicated that xylose could be used as an adjunct even when resistance mechanisms are mixed.

**Fig 6:**
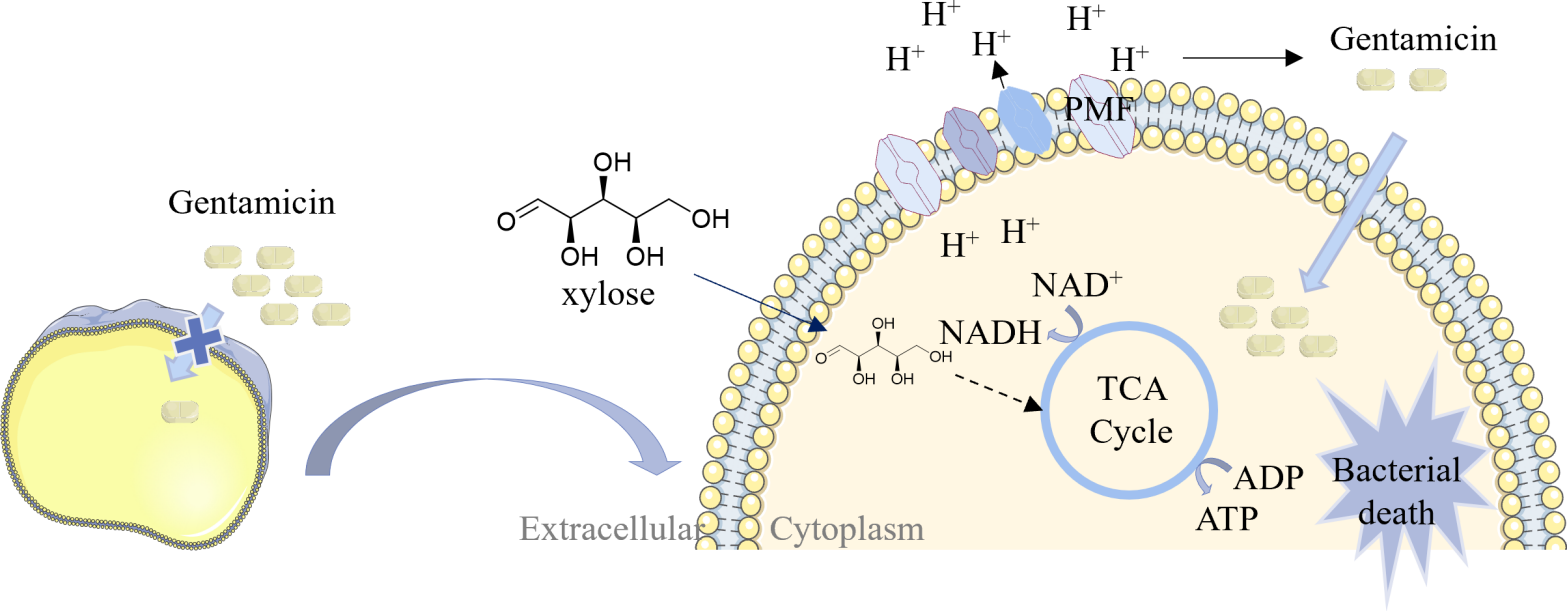
Mechanism diagram.

In conclusion, our study provided evidence that xylose not only enters but also reactivates the suppressed TCA cycle in drug-resistant *E. coli*. Xylose reprogrammed the metabolism of ECO-R_GEN_, effectively channeling carbon through glycolysis and pyruvate metabolism into the TCA cycle, leading to increased conversion of NAD ^+^ to NADH and ADP to ATP, which enhanced the uptake and bactericidal effect of gentamicin (Fig. 6). Thus, we established a link between metabolic reprogramming and restored antibiotic efficacy by xylose. Importantly, xylose is efficiently utilized by pathogens but minimally metabolized in humans. This selectivity not only extends the utility of existing antibiotics but also establishes a new strategy for exploiting host-pathogen metabolic differences to develop “pathogen-focused” adjuvants. Further assessment is needed to evaluate the potential impact of long-term xylose administration on the host microbiota to determine its clinical efficacy and safety.

## MATERIALS AND METHODS

### Strains and culture

The bacterial strains used in this study included *Escherichia coli* K12 BW 25113 (ECO-S), a knockout strain Δ*sucA* derived from ECO-S, a clinically isolated multidrug-resistant *Escherichia coli* 1 (MDR-ECO1), a clinically isolated *carbapenem-resistant Klebsiella pneumoniae* 1 (CR-KPN1), and a clinically isolated carbapenem-resistant *Pseudomonas aeruginosa* 1 (CR-PAE1); all these strains were stored in our laboratory. The MICs of multiple antibiotics against MDR-ECO1, CR-KPN1, and CR-PAE1 were shown in Table S2. Gentamicin-resistant *Escherichia coli* (ECO-R_GEN_) was obtained by serial passage of ECO-S in LB broth containing 1/2 × MIC of gentamicin until the MIC increased to 32 μg/mL. All bacteria were cultivated in 50 mL of Luria-Bertani (LB) broth at 37°C for 16–20 h (overnight bacterial cultures).

### MIC measurement

MIC was performed as previously described (35–37). According to Clinical and Laboratory Standards Institute (CLSI) guidelines, overnight bacteria cultures were diluted 1:100 into fresh Mueller-Hinton broth (MHB) medium and incubated at 37°C with shaking at 200 rpm until the OD_600_ nm reached 0.5. The bacterial suspension was then diluted with fresh MHB medium to a concentration of 5 × 10^6^ CFU/mL, and 10 μL of this suspension was added to 100 μL of MHB medium containing gradient concentrations of antibiotics in each well, ensuring a bacterial count of 5 × 10^4^ CFU in each well. To ensure accuracy, the diluted bacterial suspension was inoculated into the wells of a 96-well microplate within 5 min. The plates were then incubated at 37°C for 16 h, after which the MIC values were recorded.

### Whole-genome sequencing

For whole-genome sequencing, strains were grown to the logarithmic phase, and cell pellets were harvested by centrifugation at 14,000 × *g* and 4°C for 1 min, then rapidly frozen in liquid nitrogen. Genomic DNA was then extracted and sequenced on the Illumina platform. Quality-filtered reads were mapped to the reference genome ECO-S for variant calling. All analyses were performed using three biological replicates. The analysis focused on three susceptible (ECO-S-1/2/3) and three resistant (ECO-R_GEN_-1/2/3) strains to compare their relationships with the reference genome GCF_000005845.2 at the whole-genome level, evaluated variant differences, and screened candidate loci/genes for explaining the resistant phenotype. The analysis pipeline started with cleaned paired-end sequencing data aligned by BWA-MEM, followed by pre-processing with Samtools/GATK (sorting, deduplication, read group addition, and pair correction), joint variant detection via HaplotypeCaller, CombineGVCFs, and GenotypeGVCFs, and finally filtering with GATK VariantFiltration to construct a filtered downstream data set, removing sites with missing QD values.

### Percent survival analysis for bacteria in M9 medium

Overnight bacterial cultures were harvested by centrifugation at 5,000 × *g* for 5 min, and the supernatant was discarded. The pellet was resuspended in saline and centrifuged under the same conditions to remove the supernatant, and this process was repeated twice. Subsequently, the final pellet was resuspended in M9 medium (containing 17.1 g/L Na₂HPO₄·12H₂O, 3 g/L KH₂PO₄, 1 g/L NH₄Cl, 0.5 g/L NaCl, 10 mM sodium acetate, 2 mM MgSO₄·7H₂O, and 0.1 mM CaCl₂), with the optical density was adjusted to an OD_600_ nm value of 0.6. Five milliliter aliquots were dispensed into tubes. Metabolites and/or antibiotics were added to each tube as appropriate, and an equal-volume bacterial suspension without metabolites or antibiotics serving as the control group. The cultures were incubated at 37°C with constant shaking at 200 rpm for 6 h or the required duration. After incubation, 10-fold serial dilutions were prepared in saline, and 5 μL of each dilution was spread onto LB agar square plates. The plates were incubated at 37°C for 12 h, after which the spots were counted and multiplied by the dilution factor. Percent survival was calculated as (spots of experimental group/spots of control group) × 100% and used as the vertical coordinate value for the graph.

### Determination of inhibition zone of gentamicin and/or xylose

Overnight bacteria cultures were diluted 1:100 into 20 mL of fresh LB medium and incubated at 37°C with shaking at 200 rpm until the OD_600_ nm reached 1.0. The bacterial suspension was then diluted a further 100-fold. Meanwhile, 12.5 mL of LB agar medium was added to each Petri dish. For inoculation, 80 μL of the diluted bacterial suspension was evenly spread onto the agar surface using sterile glass beads to ensure uniform coverage. After drying for 10 min, an Oxford cup (7.8 × 6 × 10 mm) was placed in the center of each plate, and 50 μL of the test sample was added to the cup. The plates were allowed to dry for an additional 10 min and then incubated at 37°C for approximately 20 h. Following incubation, the diameter of the inhibition zone was measured and photographs were taken.

### Calculation of synergy index

The inhibition rates (inhibition rate = 1 − survival rate) were calculated from bacterial survival rate data obtained with different concentration combinations of xylose and gentamicin (three biological replicates per group) and summarized into a matrix. This matrix was then uploaded to the open-source tool Synergy Finder 3.0 (https://synergyfinder.fimm.fi). Subsequently, the ZIP model was applied to calculate the synergy scores. A synergy score greater than 10 indicated a synergistic effect (38).

### Swimming behavior test

Each 2.5 μL aliquot of overnight bacteria culture was precisely spotted onto the *Escherichia coli* identification agar (Huankai Microbial, CRM002). The plates were allowed to dry naturally for 20 min. Following this, the plates were incubated at 37°C for 24 h. After the incubation period, high-resolution photographs of the plates were taken, and the swimming diameter was measured.

### Metabolomics sample preparation, data collection, and analysis

This experiment was conducted as previously reported (12). Briefly, bacterial cultures were treated with or without xylose for 6 h following the survival rate protocol, then centrifuged at 5,000 × *g* for 5 min, and quenched with ice-cold methanol. The supernatant was discarded, and the cell pellet was resuspended in saline. Three replicates of 10 mL bacterial suspension (OD_600_ nm = 1.0) were prepared. After centrifugation at 5,000 × *g* for 5 min, the supernatant was discarded, and the cell pellet was transferred to 1.5 mL QSP tubes. Metabolite extraction was performed using ultrasonic cell disruption at an operating power of 650 W × 35%, with a pulse cycle of 2 s on and 3 s off for a total duration of 10 min. The lysate was centrifuged at 12,000 rpm for 10 min to separate the supernatant, which was then transferred to a vacuum centrifugal dryer and dried at 37°C to evaporate residual methanol. The dried samples were derivatized by adding 80 μL of a 20 mg/mL methoxylamine hydrochloride solution in pyridine, followed by incubation at 37°C for 3 h. Subsequently, 80 μL of N-methyl-N-trimethylsilyl trifluoroacetamide (MSTFA; Sigma-Aldrich) was added, and the mixture was incubated at 37°C for 30 min. Finally, 120 μL of the derivatized supernatant was transferred to a liner tube for GC-MS analysis. Each experimental group included four biological replicates.

GC-MS analysis was conducted using an Agilent 8890 gas chromatograph coupled with a 5977B mass spectrometer. The initial temperature was held at 70°C for 5 min, followed by a ramp to 270°C at a rate of 4°C/min, and a final hold at 270°C for 5 min. A 1 μL aliquot of each sample was injected in non-split mode, with the inlet temperature set to 270°C. The interface and ion source temperatures were maintained at 270°C and 230°C, respectively, while the quadrupole temperature was set to 150°C. Ionization was performed at 70 eV, and high-purity helium was used as the carrier gas at a flow rate of 1.0 mL/min. Data acquisition was performed in full-scan mode, covering a mass range of 60–600 *m*/*z*. Each experimental group included two technical replicates.

Metabolite identification was achieved by comparing chromatographic peaks and electron ionization (EI) mass spectra with the 2008 NIST database. Data normalization was performed based on the total ion count to standardize metabolite identity, retention indices, and peak areas. Statistical significance was assessed using IBM SPSS Statistics 19, with metabolites exhibiting *P* values < 0.05 considered significant. R software was employed for hierarchical clustering and heatmap visualization of metabolite profiles. Further multivariate analysis, including principal component analysis (PCA) and S-plot analysis, was conducted using SIMCA-P + 12.0 and IBM SPSS Statistics 19. Metabolic pathway analysis was performed using MetaboAnalyst 4.0 and final data visualizations were generated using GraphPad Prism 8.0.

### Quantitative real-time polymerase chain reaction

RNA extraction was performed on bacterial strains treated with or without xylose for 6 h according to the percent survival analysis method. Bacterial cells were collected by centrifugation at 5,000 × *g* for 3 min, and 10 mL of bacterial suspension was harvested. The cells were then resuspended and washed twice with saline, and the resulting pellet was retained for RNA extraction. One milliliter of Trizol was immediately added to the pellet to resuspend the cells, and the mixture was thoroughly mixed, followed by incubation at room temperature for 10 min. The mixture was then centrifuged at 12,000 × *g* at 4°C for 10 min, and the supernatant was collected and allowed to stand at room temperature for 3 min. After adding 200 μL of chloroform and vortexing for 15 s, the mixture was allowed to stand at room temperature for 3 min, followed by centrifugation at 12,000 × *g* at 4°C for 15 min. The upper 400 μL of the clear liquid was transferred to a new 1.5 mL RNase-free centrifuge tube, and an equal volume of isopropanol was added. The mixture was mixed by inverting and then allowed to stand at room temperature for 5 min, followed by centrifugation at 12,000 × *g* at 4°C for 5 min. The supernatant was discarded, and the precipitate was retained. The precipitate was washed with 1 mL of 75% ethanol and centrifuged at 12,000 × *g* at 4°C for 5 min. This process was repeated twice, and the pellet was then subjected to a final centrifugation at 12,000 × *g* at 4°C for 1 min without the supernatant. The pellet was dried at room temperature for 20 min and then dissolved in 20 μL of RNase-free water. Each 1,000 ng of RNA was reverse-transcribed using a reverse transcription kit (Promega) according to the manufacturer’s instructions.

qRT-PCR was performed using a PrimeScript RT reaction kit (Promega). The primers used for qRT-PCR were listed in Table S3, with the 16S rRNA gene serving as the internal control, and the reactions were conducted in a 96-well plate with the following reaction components: GoTaq qPCR Master Mix (2 ×) 5 μL, forward primer (10 μM) 0.2 μL, reverse primer (10 μM) 0.2 μL, cDNA template 1 μL (diluted 20 times), and ddH_2_O to a final volume of 10 μL. The reaction mixture was then subjected to amplification on a 7500 Real-Time PCR system (Applied Biosystems). The thermal cycling parameters were as follows: initial denaturation at 95°C for 2 min to activate the polymerase and pre-denature the template DNA, followed by 40 cycles of denaturation at 95°C for 15 s, annealing and extension at 60°C for 1 min. A final dissociation curve was generated by heating at 95°C for 15 s, 60°C for 15 s, and 95°C for 15 s. The data were analyzed using the software of the 7500 Real-Time PCR system, and the relative mRNA expression levels were calculated. In this experiment, a relative quantitative method was employed to analyze the data using the following formula: _ΔΔ_Ct = (Ct (target gene)-Ct (reference gene)) Sample A-(Ct (target gene)-Ct (reference gene)) Sample B, Fold Change = 2^-_ΔΔ_Ct, where Fold Change denotes the multiplicity of the expression of the target gene relative to the expression of the reference gene. Data from every two technical replicates were averaged and combined.

### NADH content determination

NADH content was measured using the EnzyChrom NADH Assay Kit (BioAssay Systems, S0175) according to the manufacturer’s instructions. Briefly, bacterial cultures were incubated with or without xylose for 6 h following the percent survival method. Then, bacteria were collected and washed three times with saline. The cell suspension was then adjusted to an OD_600_ nm of 1.0, and 4 mL of this suspension was centrifuged at 12,000 × *g* for 3 min to pellet the cells. Three parallel samples of the resulting cells were each resuspended in 200 µL of NADH extraction buffer and vortexed for 15 s and then sonicated for 3 min. The samples were then incubated in a 60°C water bath for 5 min. Thereafter, 20 μL of assay buffer and 200 µL of NAD extraction buffer were added. After vortexing for 10 s, the samples were centrifuged at 19,000 × *g* for 5 min, and the supernatant was diluted twofold and used for measurement according to the manufacturer’s instructions. A standard curve was constructed using standard solutions diluted with distilled water to final concentrations of 6, 4, 3, 2, and 1 μM.

### ATP content determination

Bacterial cultures were incubated with or without xylose for 6 h following the percent survival method, and 50 μL bacterial suspension was added to a black 96-well plate. Subsequently, 50 μL of BacTiter-Glo reagent (Promega, G8230), pre-equilibrated at room temperature for 20 min, was added to each well under low-light conditions to minimize photodegradation. The ATP content was immediately measured using a multimode microplate reader (Spectramax M2) to determine the ATP-lite linear values. Additionally, the incubated bacteria were diluted and spotted on plates to record the number of bacteria per 50 μL of bacterial suspension, which was used for ATP calculation. The standard curve was generated using a 10-fold serial dilution of the M9 standard solution, ranging from 10 pM to 1 μM (39).

### PMF content determination

For membrane potential assessment, bacterial cultures were incubated with or without metabolites for 6 h according to the survival rate protocol, pelleted by centrifugation at 5,000 × *g* for 3 min, and washed three times with M9 medium. The bacterial suspension was adjusted to an OD_600_ of 0.6, diluted 10-fold, and stained with 3 µM 3,3'-diethyloxacarbocyanine iodide at 30°C for 30 min. After centrifugation at 12,000 × *g* for 2 min, the cells were collected, resuspended in M9, and centrifuged again. This process was repeated twice. Then, the cells were diluted in sterile water and analyzed by flow cytometry (excitation: 488 nm). Bacterial populations were gated based on forward and side scatter characteristics. Membrane potential was calculated as log(10^1.5^ ×(red/green fluorescence ratio)).

### Enzyme activity assays

Bacterial cultures were treated with or without xylose for 6 h according to the percent survival method and then centrifuged at 5,000 × *g* for 5 min to remove the supernatant. The cell pellet was resuspended in saline, and three replicates of 10 mL bacterial suspensions with an OD_600_ nm value of 1.0 were collected. After centrifugation at 5,000 × *g* for 5 min to remove the supernatant, and the pellets were transferred to 1.5 mL QSP tubes. Enzyme activity was measured using commercial kits for PDH (Solarbio, BC0385), α-KGDH (Solarbio, BC0715), SDH (Solarbio, C0955), and MDH (Solarbio, BC1045). For the preparation of PDH, α-KGDH, and SDH bacterial pellets, 1 mL of Reagent I and 10 μL of Reagent II were added to each pellet. The bacterial cells were disrupted using an ultrasonic processor at 200 W, with 3 s of sonication followed by a 7 s interval, for a total duration of 5 min in an ice bath. However, for α-KGDH samples, sonication was not required; mixing was sufficient. The mixture was then centrifuged at 11,000 × *g* at 4°C for 10 min, and the supernatant was collected and kept on ice for subsequent analysis. For the preparation of NAD-MDH samples, 400 μL of extraction buffer was added to the pellet, and the bacterial or cellular pellets were disrupted using an ultrasonic processor at 200 W, with 3 s of sonication followed by a 10 s interval, repeated for 30 cycles. The mixture was then centrifuged at 8,000 × *g* with 4°C for 10 min, and the supernatant was collected and kept on ice for subsequent analysis. Enzyme activities were calculated according to the manufacturer’s instructions (40–46).

### Determination of intracellular gentamicin content

Fifty milliliters of bacterial cultures incubated with or without xylose for 6 h according to the percent survival method were centrifuged at 5,000 × *g* for 5 min. The supernatant was discarded, and the cell pellet was resuspended in saline. A 10 mL of the bacterial suspension with an OD_600_ nm value of 1.0 was collected again. The suspension was then centrifuged again at 5,000 × *g* for 5 min, the supernatant was removed, and 1 mL of saline was added to transfer the pellet to a 1.5 mL QSP tube. The pellet was centrifuged at 12,000 × *g* for 3 min to remove the supernatant. Four hundred microliters of pre-cooled saline were added to the bacterial pellet, followed by sonication (650 W × 35% power, with 2 s of sonication followed by a 3 s interval, for a total of 3 min). The supernatant containing antibiotics was obtained by centrifugation at 12,000 × *g* and 4°C for 10 min. A 50 μL of it was placed in an oxford cup, for determination according to the method described above for measuring the inhibition zone. The concentration of antibiotics was calculated according to the standard curve. For the determination of the standard curve, 300, 250, 200, 150, 125, 100, and 50 ng of gentamicin were separately added to oxford cups, and the resulting inhibition zones were measured.

### Antibacterial infection test in mice

Kunming mice (20 ± 2 g) were obtained from Changsha Tianqin Biotechnology Co., Ltd. (SCXK (Xiang) 2019-0013). Mice were challenged intraperitoneally (i.p.) with 7.2 × 10⁷ CFU of ECO-R_GEN_ or 1.8 × 10⁹ CFU of MDR-ECO1 in 200 μL LB broth. In this experiment, mice were randomly assigned to four groups (*n* = 10 per group): saline control, xylose alone (100 mg/kg in 200 μL saline, p.o.), gentamicin alone (8 mg/kg in 100 μL saline, i.m.), and a combination of xylose and gentamicin. Gentamicin was delivered via intramuscular injection into the thigh muscle. Xylose was given by gavage once daily for 3 consecutive days. Mouse mortality was monitored every 24 h over a 1-week period. During the experiment, mice were housed at 25°C under a 12 h light/dark cycle (lights on at 09:00).

For the systemic infection model, four mice were used per group. The infection and drug administration methods were identical to those described above. At 12 h post-treatment, mice were euthanized by cervical dislocation in accordance with the guidelines of the American Veterinary Medical Association, and then dissected. Kidneys and livers were aseptically removed, transferred to 1.5 mL and 5 mL sterile centrifuge tubes, respectively, and weighed. Three grinding beads and sterile saline (2 mL/g tissue) were added to each tube, and tissues were homogenized using a high-speed and low-temperature tissue grinder (Wuhan Xavier Biotechnology Co., Ltd.). Homogenates were serially diluted 10-fold in sterile saline, and 5 μL aliquots were spotted onto square LB agar plates. After incubation at 37°C for 12 h, bacterial colonies were counted to determine the bacterial load in the organs, expressed as colony-forming units per gram of tissue (CFU/g) (47, 48).

### Statistical analysis of data

Data were recorded in raw data files and plotted using GraphPad Prism 8.0. Depending on the type of experiment, statistical analysis was assessed using one-way ANOVA and two-way ANOVA, with all error bars representing mean ± standard error, with significance indicated by *P < 0.05, **P < 0.01, ***P < 0.001.

## Data Availability

All data for this article can be accessed online at https://www.scidb.cn/s/yeUvQf. The whole-genome sequencing data for the ECO-R_GEN_ have been deposited in the NCBI Sequence Read Archive (SRA) under BioProject accession number PRJNA1449439.

## SUPPLEMENTAL MATERIAL

Supplemental material aem.00731-26-s0001.pdf Fig. S1 to S6; Tables S1 to S3.

ASM does not own the copyrights to Supplemental Material that may be linked to, or accessed through, an article. The authors have granted ASM a non-exclusive, world-wide license to publish the Supplemental Material files. Please contact the corresponding author directly for reuse.
