# Supplementary material for: Xylose-induced tricarboxylic acid cycle activation resensitizes gentamicin-resistant Escherichia coli
Source: Appl Environ Microbiol. 2026 Jun 12;92(7):e00731-26. doi: 10.1128/aem.00731-26 (PMC13390390; doi:10.1128/aem.00731-26)
Supplement: Supplemental material — Fig. S1 to S6; Tables S1 to S3. [file aem.00731-26-s0001.pdf]

Supplemental Material

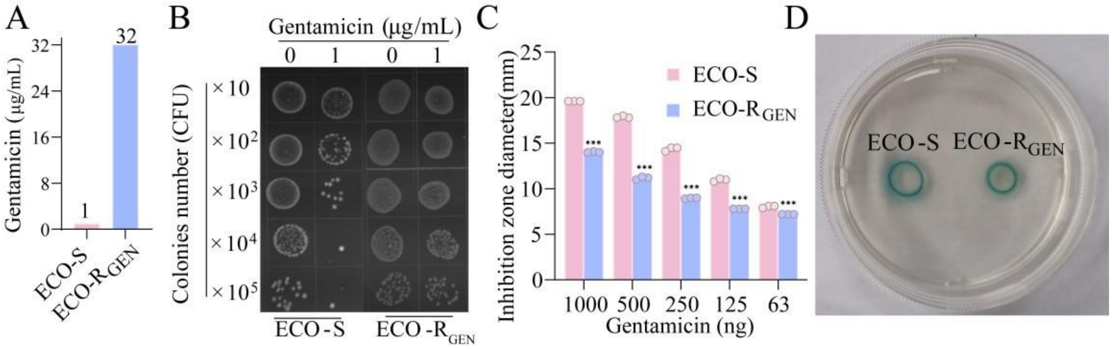

FIG S1 Analyzing the gentamicin resistance phenotype of ECO-R_{GEN}. (A) MIC of the *Escherichia coli* K12 BW25113 (ECO-S) and artificially evolved gentamicin-resistant resistant *Escherichia coli* K12 BW25113 (ECO-R_{GEN}). (B) Percent survival of ECO-S and ECO-R_{GEN} in the presence of 1 μg/mL gentamicin in M9 medium. (C) Identification of inhibition zone diameter of ECO-S and ECO-R_{GEN} strains by Oxford cup method. (D) Swarming behavior of ECO-S and ECO-R_{GEN} strains in selective *E. coil* agar medium. Experiments (A, C) were conducted with three independent biological replicates, and data. (C) was expressed as mean ± standard deviation (SD). Statistical analysis was performed using one-way analysis of variance (ANOVA) followed by post-hoc test to determine significant differences between groups (***p* < 0.01, ***p < 0.001).

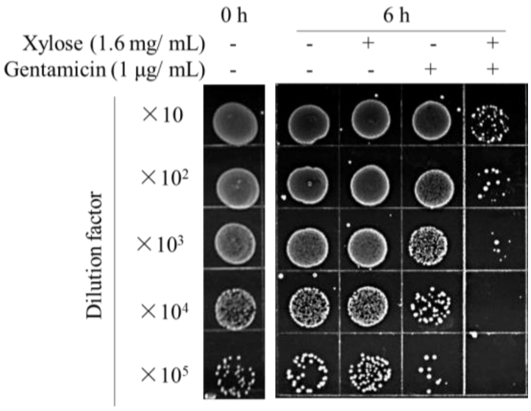

FIG S2 Spot dilution assay of ECO-R_{GEN} comparing the initial cell suspension (0 h) and the 6 h time point.

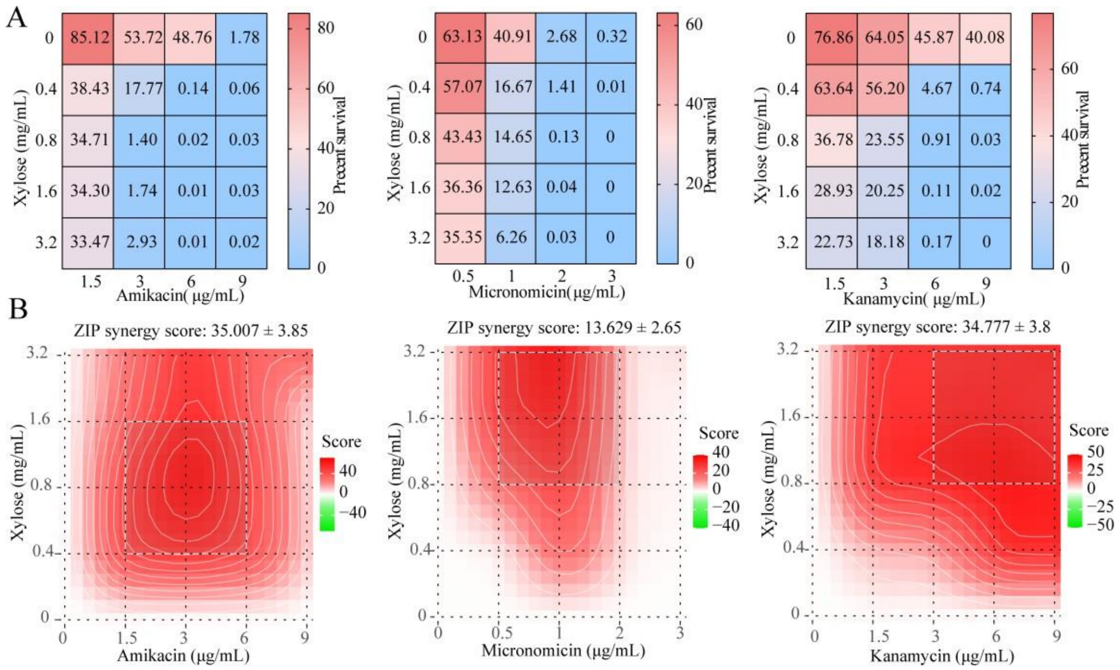

FIG S3 The effects of xylose and other aminoglycoside antibiotics on the survival rate of ECO-R$_{GEN}$. (A) Percent survival of ECO-R$_{GEN}$ in the presence of concentration gradient xylose and concentration gradient amikacin or micronomicin or kanamycin in M9 medium. (B) Synergy plots analysis of the percent survival for xylose or/and other aminoglycoside antibiotics by SynergyFinder (https://synergyfinder.org).

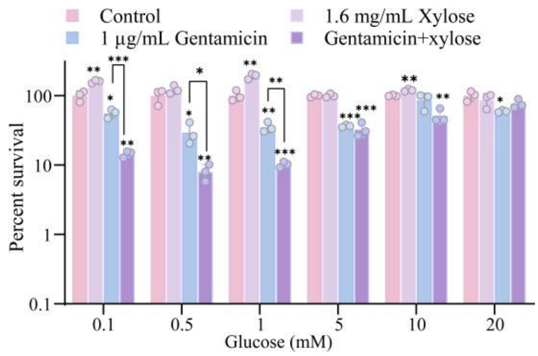

FIG S4 Percent survival of ECO-R$_{GEN}$ strains after 6 h incubation in M9 medium with glucose as the carbon source, at the specified concentrations of 1.6 mg/mL xylose and 1 µg/mL gentamicin. All experiments were conducted with three independent biological replicates, and data were expressed as mean ± SD. Statistical analysis was performed using two-way ANOVA followed by Tukey's correction to determine significant differences between groups (*$p < 0.05$, **$p < 0.01$, ***$p < 0.001$). "star(s)" above treatment with drug alone is in comparison to survival of the strain without the drug.

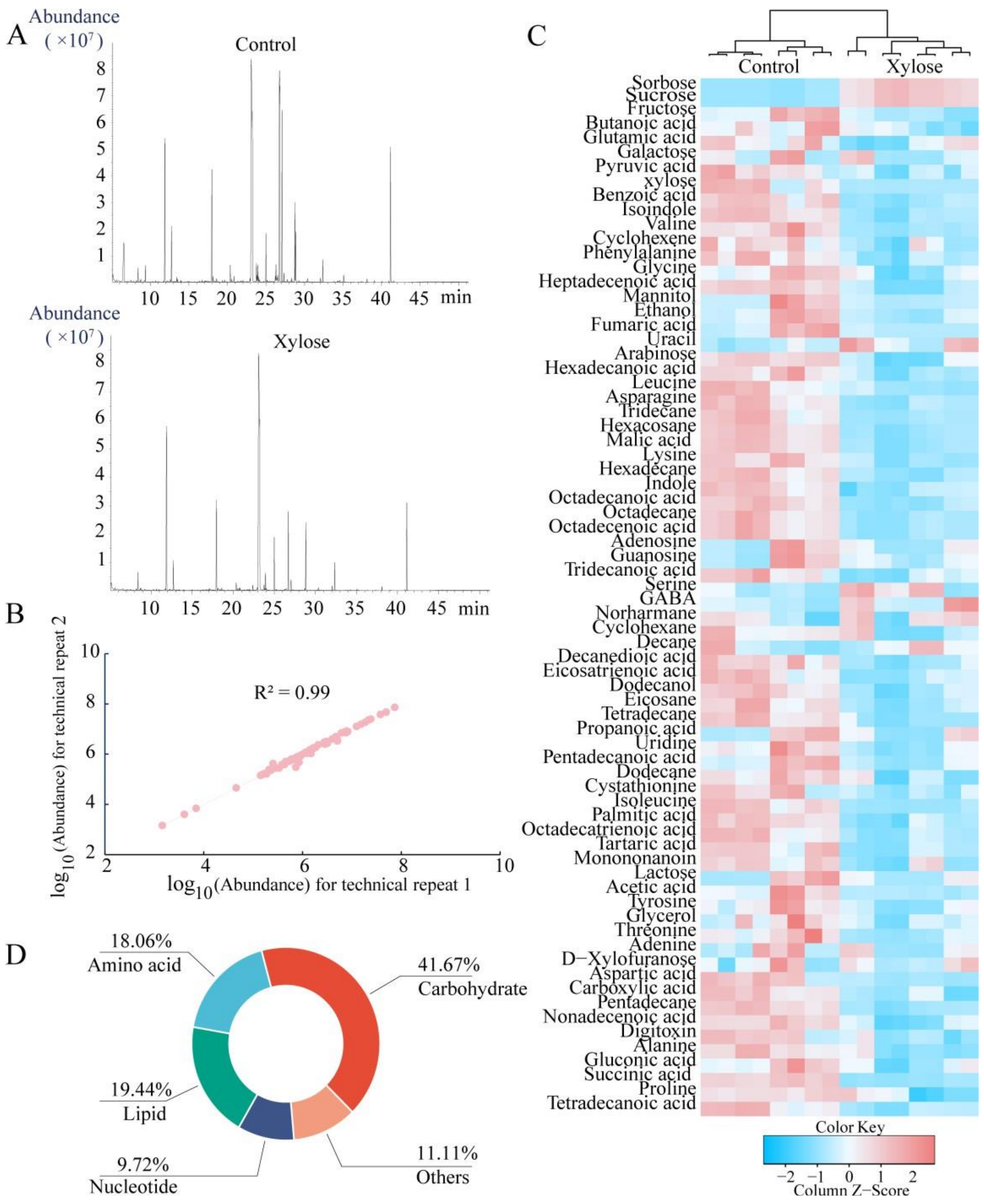

FIG S5 Metabolic profiles analysis in ECO-R_{GEN} with or without xylose. (A) Representative total ion chromatograms of metabolic abundance in ECO-R_{GEN} with or without xylose. (B) Pearson correlation coefficient between technical replicates. (C) Heat map of unsupervised hierarchical clustering of all metabolites (row). Pink color and blue color indicate increase and decrease of metabolites relative to the median metabolite level, respectively (see color scale). (D) Category of these metabolites. The percentage of each metabolite class was calculated as: class proportion (%) = (number of metabolites in each category/total number of metabolites) ×100%.

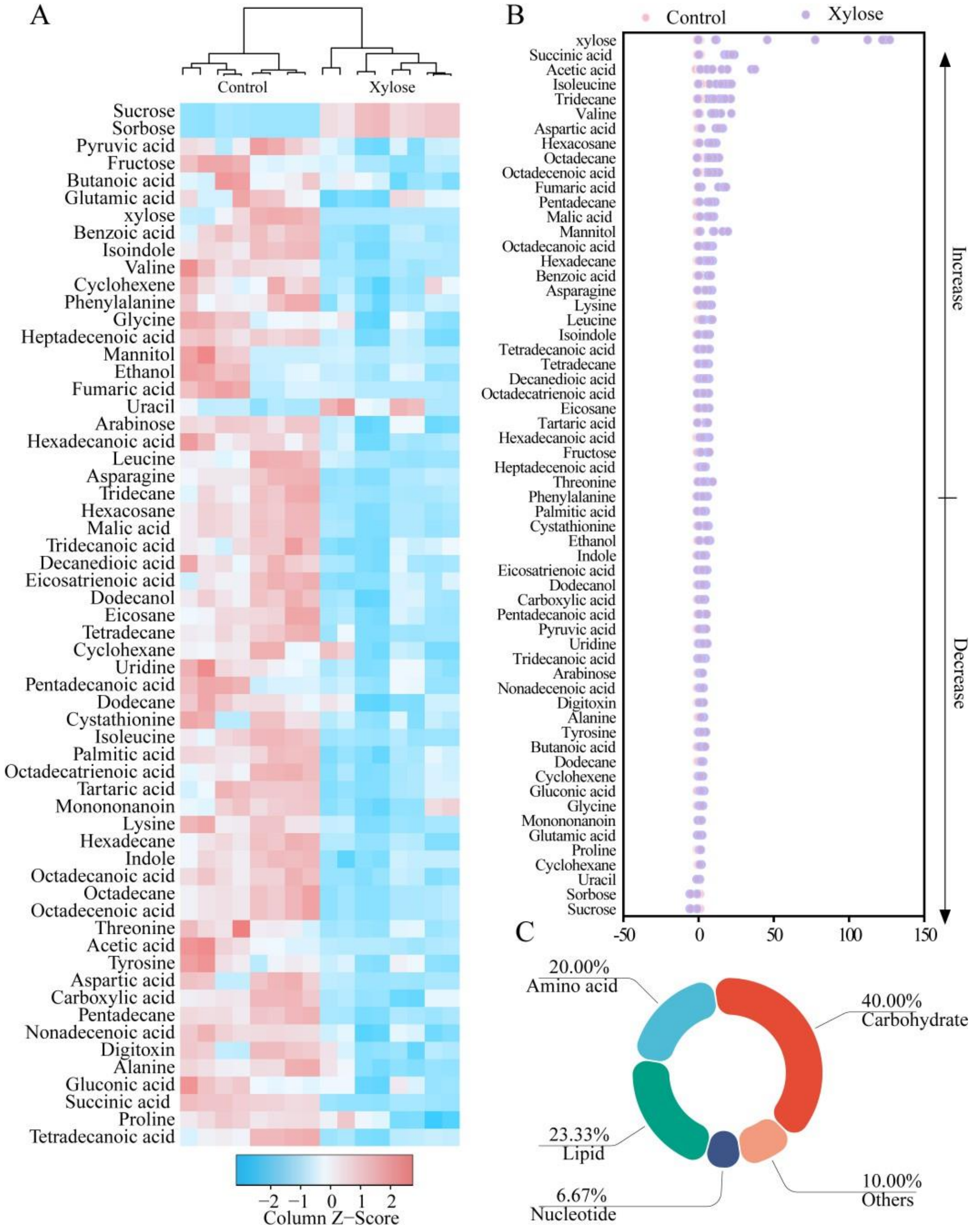

FIG S6 Differential metabolic phenotypic of ECO-R_{GEN} induced by xylose. (A) Heat map showing differential metabolites in ECO-R_{GEN} without xylose (control group) and with xylose (xylose group). Pink color and blue color indicate increase and decrease of metabolites relative to the median metabolite level, respectively (see color scale). (B) Z-score plot of differential metabolites based on control group. The data of control (left) and xylose (right) groups were separately scaled to the mean and standard deviation of control. Each point represents one metabolite in one repeat and colored by sample types. (C) Category of these differential abundances of differential metabolites. The percentage of each metabolite class was calculated as: class proportion (%) = (number of metabolites in each category/total number of metabolites) ×100%. The data (A, B) were derived from four biological replicates, with each biological replicate undergoing two technical replicates.

Supplementary table S1 Mutation sites in ECO-$R_{GEN.}$

Locus_tag	Gene	Product
b3212	*gltB*	glutamate synthase subunit
b2557	*purL*	phosphoribosylformylglycinamide synthetase
b0924	*mukB*	chromosome partitioning protein
b0726	*sucA*	subunit of E1(0) component of 2-oxoglutarate dehydrogenase
b4258	*valS*	valine--tRNA ligase
b3988	*rpoC*	RNA polymerase subunit beta
b3894	*fdoG*	formate dehydrogenase O subunit alpha
b4041	*plsB*	glycerol-3-phosphate 1-O-acyltransferase
b3956	*ppc*	phosphoenolpyruvate carboxylase
b1274	*topA*	DNA topoisomerase I
b0184	*dnaE*	DNA polymerase III subunit alpha
b1413	*hrpA*	ATP-dependent 3'->5' RNA helicase
b0098	*secA*	protein translocation ATPase
b1224	*narG*	nitrate reductase A subunit alpha
b2076	*mdtC*	multidrug efflux pump RND permease subunit
b1378	*ydbK*	putative pyruvate-flavodoxin oxidoreductase
b2231	*gyrA*	DNA gyrase subunit A
b0114	*aceE*	pyruvate dehydrogenase E1 component
b1702	*ppsA*	phosphoenolpyruvate synthetase
b1783	*yeaG*	protein kinase
b0598	*cstA*	pyruvate transporter
b3352	*yheS*	putative ATP-binding protein
b1687	*ydiJ*	D-2-hydroxyglutarate dehydrogenase
b3729	*glmS*	L-glutamine-D-fructose-6-phosphate aminotransferase
b3987	*rpoB*	RNA polymerase subunit beta
b3940	*metL*	fused aspartate kinase/homoserine dehydrogenase 2
b1049	*opgH*	osmoregulated periplasmic glucans biosynthesis protein H
b2697	*alaS*	alanine-tRNA ligase/DNA-binding transcriptional repressor
b0033	*carB*	carbamoyl-phosphate synthetase large subunit
b0584	*fepA*	ferric enterobactin outer membrane transporter
b1719	*thrS*	threonine-tRNA ligase
b0221	*fadE*	acyl-CoA dehydrogenase
b1713	*pheT*	phenylalanine-tRNA ligase subunit beta
b3407	*yhgF*	RNA-binding protein
b2114	*metG*	methionine-tRNA ligase
b2763	*cysI*	sulfite reductase, hemoprotein subunit
b3942	*katG*	catalase/hydroperoxidase HPI
b0026	*ileS*	isoleucine-tRNA ligase
b0635	*mrdA*	peptidoglycan DD-transpeptidase MrdA
b4079	*fdhF*	formate dehydrogenase H
b0732	*mngB*	alpha-mannosidase

b4058	*uvrA*	UvrABC excision nuclease subunit A
b1474	*fdnG*	formate dehydrogenase N subunit alpha
b3080	*ygjK*	glycoside hydrolase
b0167	*glnD*	protein-PII uridylyltransferase/uridylyl-removing enzyme
b0480	*ushA*	5'-nucleotidase/UDP-sugar hydrolase
b1830	*prc*	tail-specific protease
b3667	*uhpC*	inner membrane protein sensing glucose-6-phosphate
b3846	*fadB*	multifunctional enoyl-CoA hydratase

Supplementary table S2 MIC of CR-KPN1, MDR-ECO1 and CR-PAE1.

Agents	Ampicillin	Piperacillin sodium	Cefuroxime sodium	Cefotaxime	Gentamicin	Kanamycin	Tetracycline	Ciprofloxacin	Meropenem	Imipenem
Breakpoints (μg/mL)	R≥32 S≤8	R≥32 S≤8	R≥32 S≤8	R≥4 S≤1	R≥8 S≤2	R≥25 S≤16	R≥16 S≤4	R≥1 S≤0.25	R≥4 S≤1	R≥4 S≤1
CR-KPN1	>128	>128	>128	>128	≥64	64	>128	>128	256	≥128
MDR-ECO1	>128	>128	>128	>128	64	≥16	64	0.25	0.25	<0.25
Breakpoints (μg/mL)	None	R≥128 S≤ 16	None	None	R≥16 S≤4	None	None	R≥2 S≤0.5	R≥8 S≤2	R≥8 S≤2
CR-PAE1	>128	>128	>128	>128	16	>128	128	≥64	16	16

Note: refer to CLSI M100, 32nd edition, 2022.

Supplementary table S3 qRT-PCR primer used in this study.

Primer name	Forward primer	Reverse primer
16S rRNA	GTAGTCCACGCTGTAAACGA	GAATTAAACCACATGCTCCA
xylA	CTCAAAATCCTCAAACCCG	GGAAAAACTCAAATGCGACA
xylB	AATGTCTGTGATGCTGAGTG	GCCTGGGGATTATTGTGT
tktA	CGACGACACCGCAAT	GCACATCAGCAGGGAA
tktB	GCCCAGGATGAGGAA	CCAAAGAACGGGAACA
pykA	AACTTTTCTCACGGCTCGC	GGTGGATACACGGATTTTGG
ppsA	GATGTAGACAGGGTTGGG	TCAGGCTGGAAGGGAGT
pykF	TTGTTGCTGCTTCCTTTATTCG	CGGGCGTGGGTTTTTGA
aceE	AGGTCGTCTGACTCAGGAGC	CCATAGATACGGTCGGGAAC
aceF	CCGAGTTGGAAGCGTT	TGCCTTTCTTGTTGACG
ydbK	ATCTACCCTATTACCCCCA	AAAGCGCCATGCACG
lpd	CGTCGGCTGTATCCCTT	AGACCGTTGACCACTTTGA
gltA	CAAAAGCAAAACTCACCCT	GCAGCAAAATACCTTCATCAC
prpC	CGGGGCGAATGAAGTGT	GGGTCAGCGATGGTGTAAA
Icd	GAGATGGGGGTGAAGAAAA	CTGCCAGGGCGTCAGAAAT
acnA	GAAGCCGCAATGTTAG	ACGGTAGTGAATCCAGAC
acnB	TGGTTTGTGCCTCGG	TCGCCTGACGGAACACA
sucC	CGGCGGTATCGTTCGTT	GCATCCGTCAGACCTTTT
sucD	AAGCCGTTGCTGCCACT	GACCGAAACCGTAATCC
sucA	GCTGGAGCAGGAAAAAGT	TGGCGGTGGAAGAAGGTA
sucB	GAAGAGCAAAACAACGATG	AGTCAGACGACCACCCA
sdhA	AATGAGAAAGGCGAAGATG	CACGGAAGACCGAGAAG
sdhB	ACGGGCTGTATGAATGT	TCGGTATCACGGCTATC
sdhC	CTGCTGTGGCTTCTGGGTA	TTGGCGGAGCGTTTA
sdhD	TAAGCAACGCCTCCG	ACACCCCACACCACAAC
frdA	CTCAAACACACCCTCGC	GCTGCTTCCGCCTTAT
frdC	AACTGGCACCGAAAGC	GGGCAACAAACAGGATT
frdD	CGTATTCCTGTTCCTGAT	GACCGTAGAAAACCCATT
frdB	ACTAACATCCAGACCCCG	CGCTCCTTCTTACCGTG
fumA	TGGCGACGAATACAAAT	TCCACCAATAACGAACG
fumB	AACCGCCCGATGAAA	AAGCGACGCACTCCA
fumC	ATGGGGGCGATTGATG	GTTGGAACTTTGGCTTTTGT
fumD	GGACAAAAGAGGATGAAC	AATACGCCTGCGATAA
fumE	AACGAACAGGATGAAACG	ACGGAGAAATCAGCGAA
mdh	GTTACCATTCTGCCGCT	GATAGATTTACGCTCTTCCAC
mqo	CAATGGCAGGCTACG	CTTCTTTTTGAACGGGT
